# Supplementary material for: Understanding the Working Mechanism of the Novel HKUST-1@BPS Composite Materials as Stationary Phases for Liquid Chromatography
Source: Polymers (Basel). 2022 Mar 28;14(7):1373. doi: 10.3390/polym14071373 (PMC9002510; doi:10.3390/polym14071373)
Supplement: Supplementary file 1 [file polymers-14-01373-s001.zip › polymers-1640022-Supplementary.pdf]

polymers

Supplementary Information

Understanding the Working Mechanism of the Novel HKUST-1@BPS Composite Materials as Stationary Phases for Liquid Chromatography

Bulat R. Saifutdinov [1,2,*], Vera I. Isaeva [3,*], Vladimir V. Chernyshev [1,2], Vadim V. Vergun [3], Gennady I. Kapustin [3], Yulia P. Ivanova [1], Mikhail M. Ilyin [4], Olga P. Tkachenko [3], Aleksey K. Buryak [1] and Leonid M. Kustov [2,3,5]

1. HPLC examinations of the HKUST-1@BPS Composite Materials

2. XRD examinations of the HKUST-1@BPS Composite Materials

3. DRIFTS examinations of the HKUST-1@BPS Composite Materials

1. HPLC examinations of the HKUST-1@BPS Composite Materials

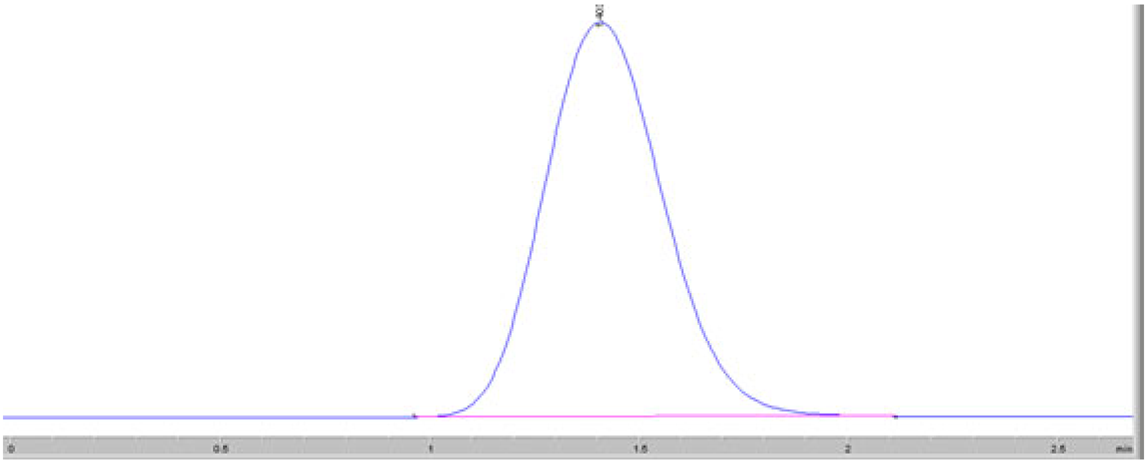

Figure S1. Output curve (chromatogram) recorded during the elution of benzene at 65°C through the porous layer of the HKUST-1@BPS composite material (sample **1**).

2. XRD examinations of the HKUST-1@BPS Composite Materials

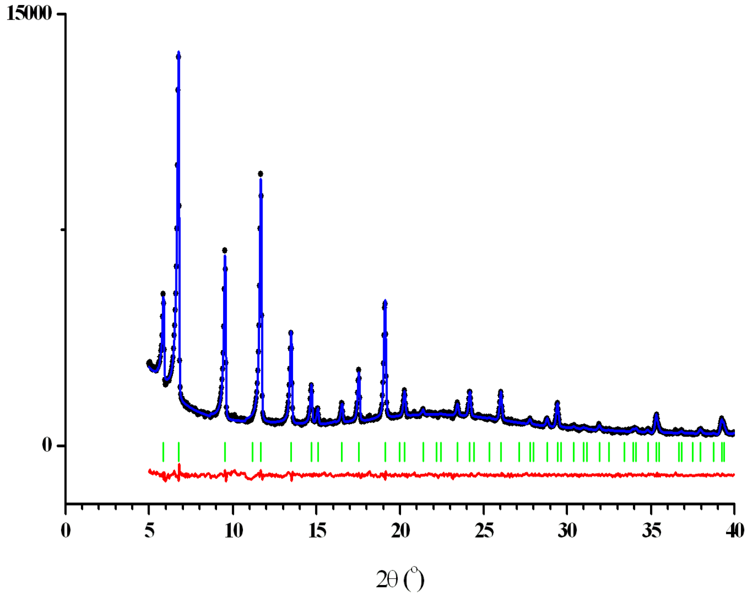

Figure S2. The result of the Pawley fitting for **1** (c^2 = 1.8, cubic unit cell parameter *a* = 26.270(2) Å, sp. gr. *Fm-3m*), showing the experimental pattern (black dots), calculated (blue) and difference (red) curves. Vertical green bars denote calculated peak positions.

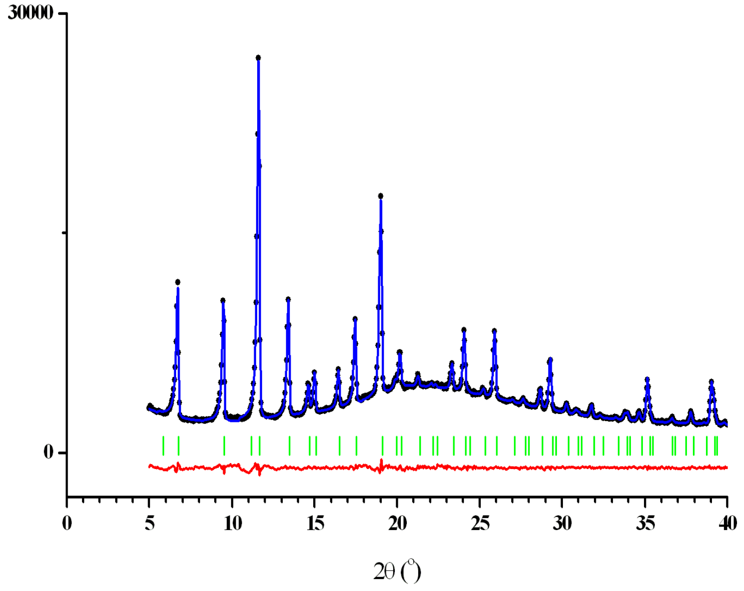

Figure S3. The result of the Pawley fitting for **2** (c^2 = 2.4, cubic unit cell parameter *a* = 26.392(2) Å, sp. gr. *Fm-3m*), showing the experimental pattern (black dots), calculated (blue) and difference (red) curves. Vertical green bars denote calculated peak positions.

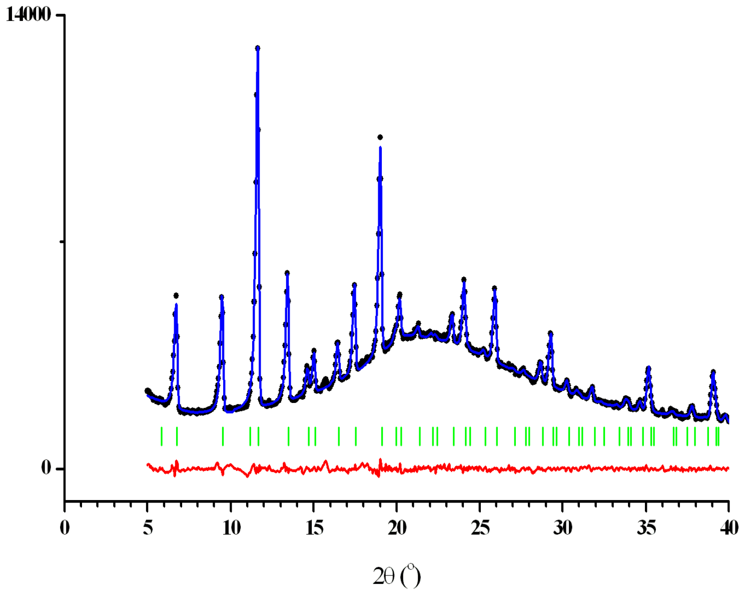

Figure S4. The result of the Pawley fitting for **3** (c^2 = 1.7, cubic unit cell parameter *a* = 26.397(2) Å, sp. gr. *Fm-3m*), showing the experimental pattern (black dots), calculated (blue) and difference (red) curves. Vertical green bars denote calculated peak positions.

3. DRIFTS examinations of the HKUST-1@BPS Composite Materials

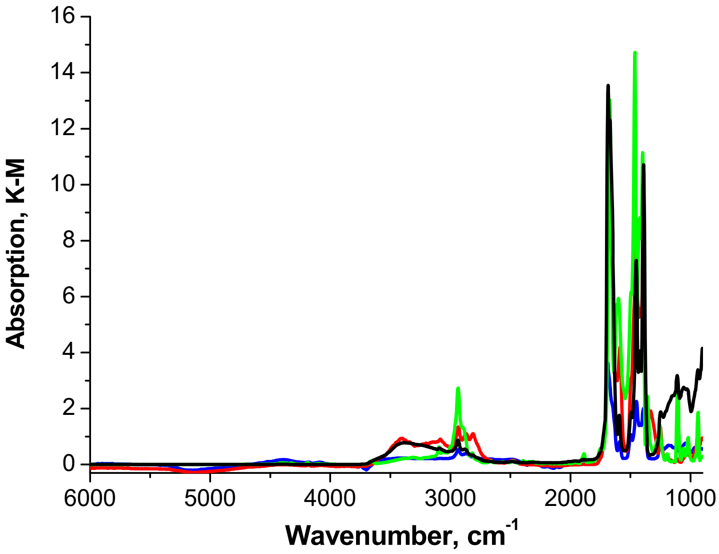

Figure S5. Survey DRIFT spectra of the **1** (black), **2** (red), **3** (green) and HKUST-1*mw* (blue) materials.

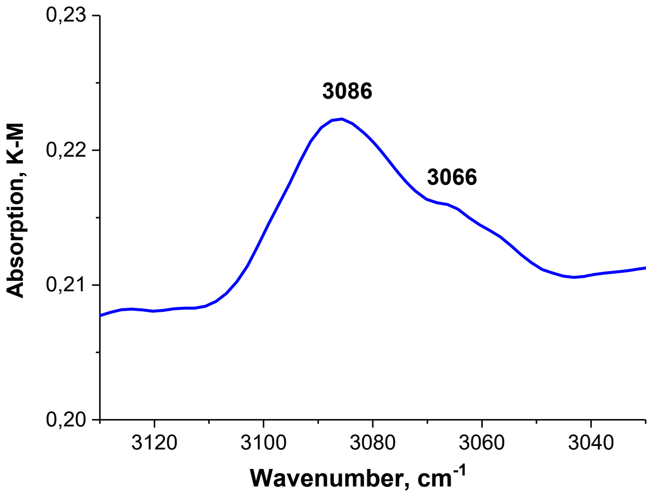

Figure S6. DRIFT spectrum of the composite **3** in the frequency range of 3130–3030 cm^{-1}.
